# Effects of a microbial restoration substrate on plant growth and rhizosphere bacterial community in a continuous tomato cropping greenhouse

**DOI:** 10.1038/s41598-020-70737-0

**Published:** 2020-08-13

**Authors:** Xuefang Zheng, Ziran Wang, Yujing Zhu, Jieping Wang, Bo Liu

**Affiliations:** 1 Agrobiological Resource Research Institute, Fujian Academy of Agriculture Sciences, Fuzhou, 350003 China; 2 Department of Biochemistry and Biotechnology, School of Life Sciences, Xiamen University, Xiamen, 361102 China

**Keywords:** Applied microbiology, DNA sequencing

## Abstract

Continuous cropping of tomato is increasingly practiced in greenhouse cultivation, leading to several soil-related obstacles. In this study, a type of microbial restoration substrate (MRS) was used to amend soils from the re-cropping of tomato for 8 years under greenhouse-cultivated conditions. Two treatments were established: using 1,500 kg hm^−2^ of MRS to amend soil as treatment (TR), and non-MRS as control (CK). The severity of bacterial wilt (BW), soil properties and rhizobacterial community composition under two different treatments were compared. The application of MRS led to an average 83.75% reduction in the severity of BW, and significantly increased the plant height, root activity and yield. Meanwhile, soil pH, soil organic contents (SOC), total nitrogen (TN) and exchangeable calcium were significantly increased (*P* < 0.05) by MRS treatment. Illumina-MiSeq sequencing analysis of the 16S rRNA genes revealed that MRS increased the diversity of the tomato rhizobacterial community. The relative abundances of Proteobacteria, Actinobacteria and Bacteroidetes were enhanced, whereas those of Acidobacteria, Chloroflexi, TM7 and Firmicutes were decreased by MRS. The redundancy analysis (RDA) revealed that the severity of tomato BW was negatively correlated with the relative abundances of Actinobacteria, Bacteroidetes and Proteobacteria, but positively correlated with those of Gemmatimonadetes, Firmicutes and Acidobacteria. In addition, the effects of MRS on rhizobacterial metabolic potentials were predicted using a Kyoto Encyclopedia of Genes and Genomes (KEGG) database, implying that MRS could significantly increase nitrogen metabolisms and reduce carbon metabolism. Together, our results indicated that the use of MRS could reestablish soil microbial communities, which was beneficial to plant health compared with the control.

## Introduction

Planting practice is one of the key factors that impact crop yield and agricultural product quality^1,2^. In recent years, the main planting practices of tomato (*Lycopersicon esculentum* Mill.), namely long-term continuous cropping, have caused a series of microecological imbalance problems^3^. The tomato field soils have gradually been transformed from high-fertility to low-fertility, and the soil pH has been changed from neutral to acidic, which has led to serious soil-born diseases, such as bacterial wilt (BW)^4^.

Soil microorganisms are vital for soil ecosystems, as they dominate the cycling of nutrients, the decomposition of organic matter, and the maintenance of soil fertility^5,6^. An appropriate community population, as well as high microbial activity and diversity, are critical for maintaining the sustainability and productivity of soil ecosystems^7,8^. Recently, many of studies had focused on the disruption of soil microbial communities under long-term continuous cropping system of tomato^9^, cucumber^10^, and vanilla^2^. Mo et al. had reported that soil bacterial population was a sensitive indicator of continuous cropping of tomato^9^. Rhizosphere is the zone of soil strongly influenced by plant roots^11^. The rhizobacterial community is importance both to plant growth and plant health^12^. Understanding soil rhizobacterial community is necessary to facilitate soil improvements under continuous cropping.

Soil amendments have been widely used to improve successive crop soil and reduce the severity of soil-born diseases^13^. Zhang et al. demonstrated that rice straw biochar application could reduce the incidence and severity of tobacco BW^14^. Rock dust additions^15^ and soil amendment with urea and calcium oxide^16^ have been reported to be effective for controlling tomato BW. In an earlier study by our group, a type of microbial restoration substrate (MRS, consisting of 33% chaff, 33% coir, 34% rice straw, and abundant microorganisms, see below) was used to amend the successive tomato soil in an open field and proved to be effective^17^. However, application efficiency of MRS in a continuous tomato cropping greenhouse and its potential mechanisms remain unknow.

In this study, the influence of MRS on rhizosphere bacterial community composition and diversity was proposed, thereby exerting significant impacts on soil productivity and health. In the light of the above considerations, bacterial community structures of tomato rhizosphere soils under MRS treatment and non-MRS treatment (CK) were compared using 16Sr RNA high-throughput sequencing technology. The main objective was to evaluate the effects of MRS on soil rhizobacterial community.

## Materials and methods

### Soil restoration field trials

The soil restoration field experiments were conducted from September 2017 to January 2018 at Mayu County, Ruian City, Zhejiang Province, in southeastern of China (27° 77′ N, 120° 45′ E). Ruian City is located in the subtropical marine climate, with an average annual temperature of 17.9 °C. A tomato greenhouse undergoing 8 years of continuous cropping was selected for the experimental field, with outbreaks of BW in the past 2 years.

The tomato (cv. Hongbaoshi) seeds were sown in 32-hole plugs filled with commercial peat compost on September 18, 2017, and were grown in a greenhouse at 25–30 °C, 85–100% relative humidity. The seedlings were transplanted into plastic greenhouses after 1 month, and the plants were grown at temperatures ranging between 26 and 36 °C. The experimental field soils were treated with MRS on October 15, 2017. MRS was jointly produced by the Fujian Academy of Agriculture Sciences (FAAS) and Xiamen Jiang Ping Biological Co., Ltd., China (XJPBC), using a microbial fermentation bed system^18^. The manufacturing processes of MRS were as follows: the litters (33% chaff, 33% coir and 34% rice straw) were added onto the pig microbial fermentation bed; the aerobic fermentation was conducted for 20 days by ploughing the litters mixing with pig manures one time per day; then, the upper 20 cm litters were removed to produce MRS by drying, crushing, screening and packaging^17^. The dominant bacterial genera of MRS were *Granulicella* (3.31%), *Acidothermus* (3.20%) and *Rhodanobacter* (1.27%) (NCBI accession number: SRP144025). The physiochemical characteristics of MRS were as follows: pH 7.82, bulk density 0.12, total porosity 72.36%, water-filled pores 80.70%, aerial pores 3.65%, the soil organic carbon (SOC) 145.12 g kg^−1^, total nitrogen (TN) 0.46%, total phosphorus (TP) 0.32%, total potassium (TK) 1.65%, and exchangeable calcium 28.54 g kg^−1^.

Two treatments were established: (1) soil amendment with 1,500 kg hm^−2^ MRS (TR) and (2) the control with non-MRS (CK). Randomized block designs and triplicate plots were conducted in the experiments. Each plot consisted of 18-m-long rows, spaced 0.4 m apart, corresponding to a total 80 m^2^ plot area. The distance between adjacent plots was 0.8 m (Fig. S1). For TR, 4.38 kg of MRS was applied as soil amendment in each plot 30 days beftor tomato planting. Then, 600 kg hm^−2^ of compound fertilizer was applied as base fertilizer with a ratio 1.5:1:3 of N:P_2_O_5_:K both in TR and CK treatments 1 day beftor tomato planting. In addition, 100 kg hm^−2^ of K fertilizer was applied at fruit setting and ripening stage, respectively. The tomato plants were irrigated with water to maintain a proper moisture level of approximately 90% of water holding capacity.

### Soil sampling, disease scoring and biological investigations

The rhizosphere soil (attached to the primary and lateral roots) samples were collected by the five-spot-sampling method after application of MRS on days (d) 0, 30, 60, 90 and 120 in triplicate plots (Fig. S1). The plants were removed gently from the farmland using a spade, the loosely soils were removed through vigorous shaking or tapping and the adherent soils attached to root were collected using a brush. Then, soil samples were sieved (2-mm mesh) to remove plant debris and partitioned into two sub-samples, one for bacterial diversity detection and the other for biochemical properties tests. SOC was measured by the Walkley–Black method^19^, TN was determined by the Kjeldahl method^20^ and TK was determined using an atomic absorption spectropotometer after wet digestion of soil sample with NaOH^21^, TP was determined by alkaline digestion followed by molybdate colorimetric measurement^22^ and the exchangeable calcium was determined using atomic absorption spectrophotometry after extracting with ammonium acetate^23^. The soil pH was measured using a 1:2.5 (w:v) soil:water ratio. The physiochemical characteristics of original soil were as follows: pH 5.57, SOC 26.57 g kg^−1^, TN 0.13%, TP 0.17%, TK 1.67%, and exchangeable calcium 4.11 g kg^−1^.

The disease severity and control efficiency were recorded at the time of the soil sampling. Based on the wilt severity, tomato BW was empirically categorized into five grades: 0, no wilting; 1, 1–25% wilting; 2, 26–50% wilting ; 3, 51–75% wilting; and 4, 76–100% wilting or death of the entire plant^24^. The disease incidence, control efficiency, and disease severity index (DSI) were calculated as follows:

$$ \begin{gathered} {\text{Disease incidence }}\left( {{\text{DI}}} \right) = \sum \left( {{\text{number of diseased plants}}/{\text{total number of plants investigated}}} \right) \hfill \\ {\text{Control efficiency }}\left( {{\text{CE}}} \right) \, = { 1}00\% \, \times \, \left( {{\text{DI of control}} - {\text{DI of treatment}}} \right) /{\text{DI of control}} \hfill \\ \end{gathered} $$

DSI = (4A + 3B + 2C + 1D)/N × 100, where A represents the number of plants in grade 4, B represents the number of plants in grade 3, C represents the number of plants in grade 2, D represents the number of plants in grade 1, and N represents the total number of plants^25^.

At harvest stage, some biological characteristics were investigated, including plant height, root activity and yield. For each treatment, 30 plants were selected using five-spot-sampling method to investigate the plant height. The tomato roots of TR and CK were collected (10 g) and were measured using 2,3,5-triphenyl tetrazolium chloride (TTC) reduction method^26^. The tomato fruits of each treatment were weighed separately at per harvest time and the yields were calculated.

### Soil DNA extraction and sequencing

The soil DNA was extracted using a DNA extraction kit (Mo Bio Laboratories Inc., Carlsbad, CA). The concentrations were measured using a NanoDrop 2000 spectrophotometer (NanoDrop Technologies, Thermo Fisher Scientific, Waltham, MA). According to the concentration, the DNA was diluted to 1 ng/µL using sterile water. The V4 hypervariable regions of the bacterial 16S rRNA gene were amplified using 515F (5*′*-GTG CCA GCM GCC GCG GTA A-3*′*) and 806R (5*′*- GGA CTA CHV GGG TWT CTA AT-3*′*) primers. The PCR amplification was performed as follows: 5 min at 98 °C for initial denaturation; followed by 30 cycles of 98 °C for 30 s, 55 °C for 30 s, and 72 °C for 30 s, with a final extension at 72 °C for 5 min. The PCR products were purified with Agencourt AMPure Beads (Beckman Coulter, Indianapolis, IN) and quantified using the PicoGreen dsDNA Assay Kit (Invitrogen, Carlsbad, CA). The sequencing procedure was performed using the Illumina Miseq Platform at Personal Biotechnology Co., Ltd. (Shanghai, China).

Paired-end reads were merged using FLASH (V1.2.7, https://ccb.jhu.edu/software/FLASH/) to obtain raw tags. The raw Fastq files were de-multiplexed and quality-filtered with QIIME 2 according to previously published criteria^27^. The chimera sequences were detected and removed using USEARCH software (https://www.drive5.com/usearch/manual/uchime algo.html).

### Bioinformatics analysis

A sequence analysis was performed by UPARSE software (V7.0, https://drive5.com/uparse/). The sequences with ≥ 97% similarity were assigned to the same OTUs. The taxonomic information for each OTU was annotated by the RDP classifier (V2.2, https://sourceforge.net/projects/rdp-classifier/). The soil bacterial community diversity was analyzed using Chao1, ACE, and Shannon diversity indices. The Chao1 and ACE indices are commonly used to characterize species abudance, and Shannon diversity index accounts for both abudance and evenness of species present. All of these indices were calculated with the QIME 2 and were displayed with R software (V3.2.0). The effect of MRS on rhizobacterial metabolic potentials were predicted using PICRUSt software^28^ based on KEGG (Kyoto Encyclopedia of Genes and Genomes) database^29^ and the significance was calculated according to *p*-value (< 0.05)^30^. The ralationship between rhizobacteria at the level of phylum and metabolic pathways in KEGG was analyzed using R software (V3.2.0). All the sequences are available in the NCBI Sequence Read Archive (SRA) database under the accession number SRP136406.

### Statistical analyses

The weighted UniFrac distance metric was employed for the principal component analysis (PCA). The heatmap of relative abundance of bacteria phyla in each sample was created through R software (V3.2.0). A redundancy analysis (RDA) was performed using Canoco 5.0 (Cabit Information Technology Co., Shanghai, China) to evaluate the relationships among bacterial communities, soil properties and the severity of BW. Data on plant biological characteristics, soil physicochemical properties, the disease incidence and control efficiency were analyzed using the SPSS v20.0 (SPSS Inc., Chicago, IL). Mean comparison were conducted using Tukey’s HSD (honest significant difference) multiple range test. The *P* values of < 0.05 were considered significant.

## Results

### Disease severity of tomato BW

DSI of tomato BW was significant difference (*P* < 0.05) between the TR and CK (Table 1). Compared to the CK, MRS significantly decreased the values of DSI with 100%, 83.61%, 64.73% and 79.62% after 30, 60, 90 and 120 days application, respectively. The control efficiencies of MRS application after 30, 60, 90 and 120 days were 100, 84.91, 66.67, and 82.50%**,** respectively. These results indicated that MRS could suppress tomato BW.

**Table 1:** Efficacy of a microbial restoration substrate for control tomato bacterial wilt at different time period (mean ± SD).

Treatment	30 days		60 days		90 days		120 days	
	DSI	CE	DSI	CE	DSI	CE	DSI	CE
TR	0b*	100	4.17 ± 1.84b	84.91 ± 3.82	21.88 ± 6.32b	66.67 ± 4.32	29.63 ± 5.90b	82.50 ± 2.73
CK	5.55 ± 1.51a	–	25.44 ± 8.31a	–	62.04 ± 9.03a	–	145.37 ± 32.43a	–

Data are means ± standard deviation (n = 3).

*TR* microbial restoration substrate treatment, *CK* non-microbial restoration substrate control, *DSI* disease severity index, *CE* control efficiency (%).

*Values within a row followed by the same letter are not significantly different at *P* ≤ 0.05.

### Biological characteristics of tomato plant with and without MRS treatments

MRS significantly increased plant height (184.50 cm), root activity (53.72 µg g^−1^ h^−1^) and yield (171,664 kg hm^−2^), comparing with CK for 156.29 cm of plant height, 21.74 µg g^−1^ h^−1^ of root activity and 134,258 kg hm^−2^ of yield (Table 2). Specifically, the root activity was extremely increased by MRS with 147.10%.

**Table 2:** The effect of a microbial restoration substrate on tomato plant height, root activity and yield (mean ± SD).

Treatment	Plant height (cm)	Root activity (µg g^−1^ h^−1^)	Yield (kg ha^−1^)
TR	184.50 ± 3.56a*	53.72 ± 2.01a	171,664.33 ± 89.32a
CK	156.29 ± 2.27b	21.74 ± 3.41b	134,285.33 ± 62.64b

Data are means ± standard deviation (n = 3).

*TR* microbial restoration substrate treatment, *CK* non- microbial restoration substrate control.

*Values within a row followed by the same letter are not significantly different at P ≤ 0.05.

### Physicochemical properties of tomato rhizosphere soils with and without MRS treatments

Compared to CK, the application of MRS significantly increased soil pH and the contents of soil SOC, TN, TP and exchangeable calcium (*P* < 0.05). For example, compared to CK120 days, the contents of SOC, TN, TP and exchangeable calcium in TR120 days were increased by 257.51%, 163.63%, 20.00% and 339.40%, respectively. However, TK concentration had no significant change in the two treatments, except for 30 days sample (Table 3).

**Table 3:** Effects of a microbial restoration substrate on soil properties in the tomato rhizosphere.

Treatment	30 days						60 days					
	pH	SOC (gkg^−1^)	TN (%)	TP (%)	TK (%)	EC (cmol kg^−1^)	pH	SOC (gkg^−1^)	TN (%)	TP (%)	TK (%)	EC (cmol kg^−1^)
TR	7.30 ± 0.10a	93.22 ± 2.70a	0.37 ± 0.02a	0.24 ± 0.01a	1.69 ± 0.06a	21.70 ± 0.43a	7.17 ± 0.06a	89.26 ± 3.46a	0.35 ± 0.01a	0.22 ± 0.02a	2.11 ± 0.02a	20.72 ± 1.77a
CK	5.27 ± 0.06b	25.23 ± 0.5b	0.12 ± 0.01b	0.17 ± 0.01b	1.65 ± 0.02b	4.32 ± 0.18b	5.03 ± 0.06b	24.91 ± 1.5b	0.12 ± 0.02b	0.17 ± 0.01b	2.10 ± 0.02a	4.71 ± 0.34b

Treatment	90 days						120 days					
	pH	SOC (gkg^−1^)	TN (%)	TP (%)	TK (%)	EC (cmol kg^−1^)	pH	SOC (g kg^−1^)	TN (%)	TP (%)	TK (%)	EC (cmol kg^−1^)
TR	7.09 ± 0.01a	87.80 ± 2.19a	0.34 ± 0.02a	0.21 ± 0.02a	2.09 ± 0.03a	20.82 ± 1.11a	7.08 ± 0.05 c	85.41 ± 3.18a	0.29 ± 0.02a	0.18 ± 0.02a	2.08 ± 0.01a	20.52 ± 2.32a
CK	4.70 ± 0.10b	25.11 ± 1.9b	0.11 ± 0.01b	0.16 ± 0.01b	2.14 ± 0.02a	4.85 ± 0.11b	4.52 ± 0.20g	23.89 ± 2.3b	0.11 ± 0.01b	0.15 ± 0.01b	2.12 ± 0.02a	4.67 ± 0.37b

Data are means ± standard deviation (n = 3).

*SOC* soil organic carbon, *TN* total nitrogen, *TP* total phosphorus, *TK* total potassium, *EC* exchangeable calcium.

*Values within a row followed by the same letter are not significantly different at *P* ≤ 0.05.

### Correlation between soil properties and plant biological characteriastics

A correlation analysis was conducted between soil properties and plant biological characteristic. The result showed that soil pH, the SOC, TN, TP and exchangeable calcium contents were positively correlated, while TK concentration was negatively correlated with plant height, root activity and yield (Table S1). Particularly, soil pH and the SOC had extremely significant correlation with plant height, root activity and yield (*P* < 0.01).

### Bacterial community abundance, diversity and structure

**Figure 1:**
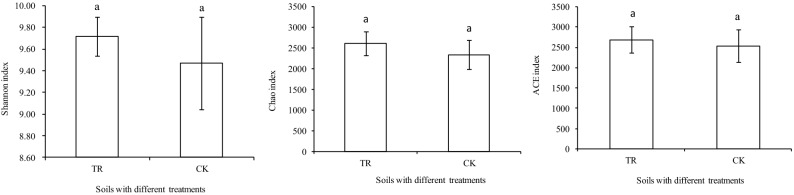
Shannon (**A**), Chao (**B**) and ACE index of soil samples with different treatments. “TR” represents microbial restoration substrate treatment; “CK” represents non-microbial restoration substrate treatment.

After quality and chimera filtering, a total of 603,451 high-quality 16S rRNA gene sequences were generated from test soil samples. These high-quality sequences were clustered into 156,807 OTUs at 97% sequence similarity. The species richness was reflected by the Shannon, Chao1, and ACE indices, which were all higher in the rhizosphere soil of TR than in that of CK (Fig. 1).

**Figure 2:**
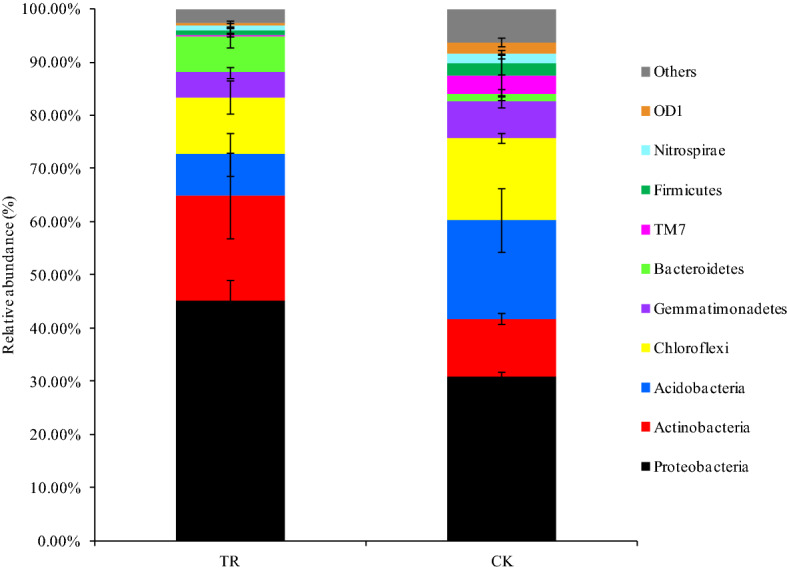
Bar chart of bacterial community composition at phylum level with abundance > 0.1%. The remaining groups with less abundance are classified into “Others”. “TR” represents microbial restoration substrate treatment; “CK” represents non- microbial restoration substrate treatment.

The dominant phyla present among all the bacterial communities were Proteobacteria, Actinobacteria, Acidobacteria, and Chloroflexi. Compared to CK, TR showed a higher relative abundance of Proteobacteria, Actinobacteria and Bacteroidetes, and a reduced relative abundance of Acidobacteria, Chloroflexi, TM7 and Firmicutes (Fig. 2). For the relative abundace of Actinobacteria, the difference between TR and CK reached to a significant level (*P* < 0.05), and for those of Proteobacteria, Acidobacteria, Chloroflexi, Gemmatimonade, Bacteroidetes, and OD1, the differences between TR and CK reached to an extremely significant level (*P* < 0.01).

**Figure 3:**
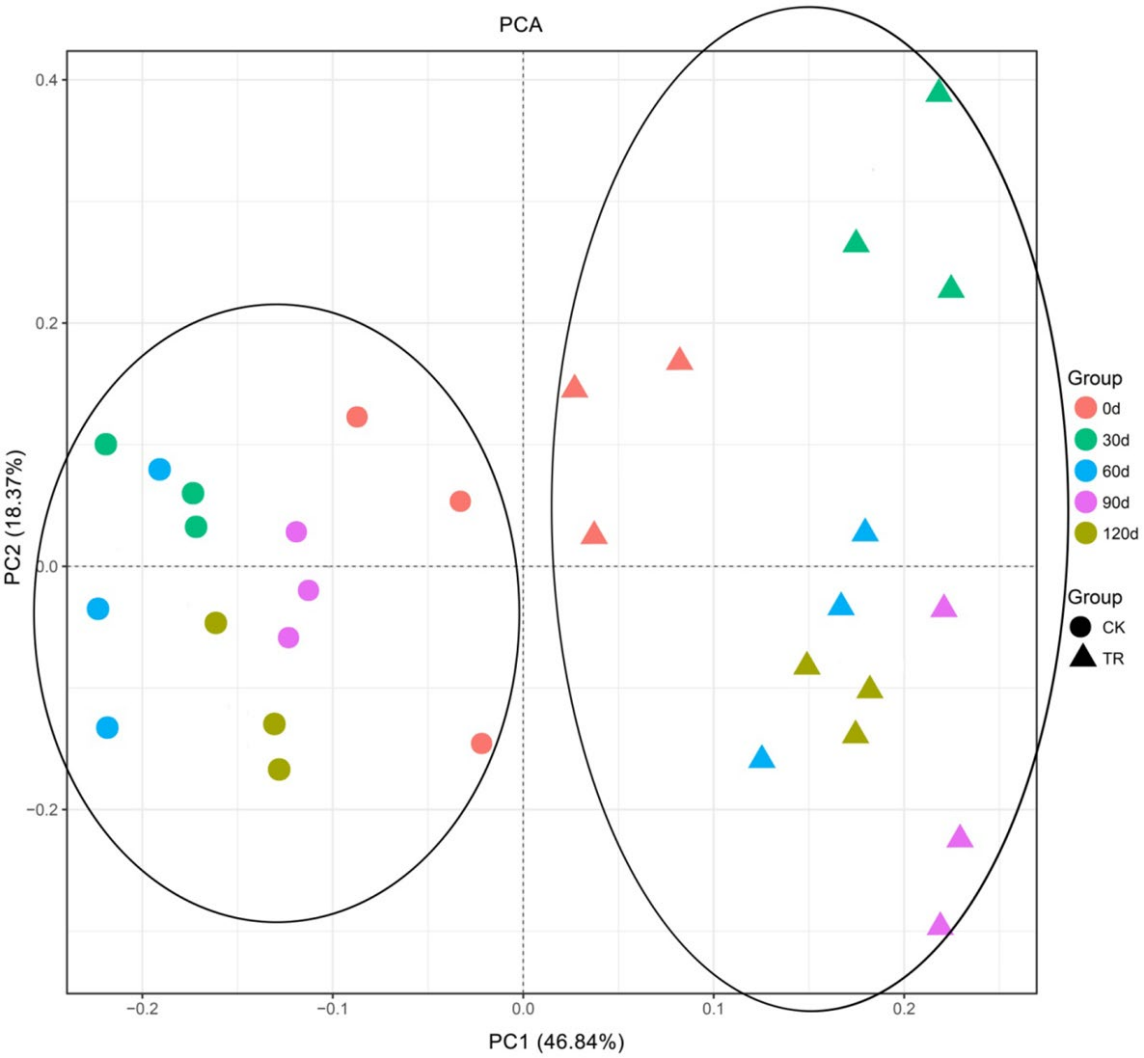
Principal component analysis (PCA) of bacterial communities in tomato rhizosphere soils with and without microbial restoration substrate treatments.

Based on the PCA supported by Euclidean distance, the samples subjected to TR and CK treatments could be separated into two groups in the PCA plot (Fig. 3). The TR samples were gathered on the right of the plot along the first principal component (PC1), and the CK samples were grouped to the left of the PC1. At the 0 day time point, TR0 day and CK0 day were located near the origin of the axis. The rhizobacterial community was significantly changed by MRS after application 30 days, which was positively correlated with the PC1 and PC2. At different time periods, the change in the rhizobacterial community for CK was less than that for TR.

**Figure 4:**
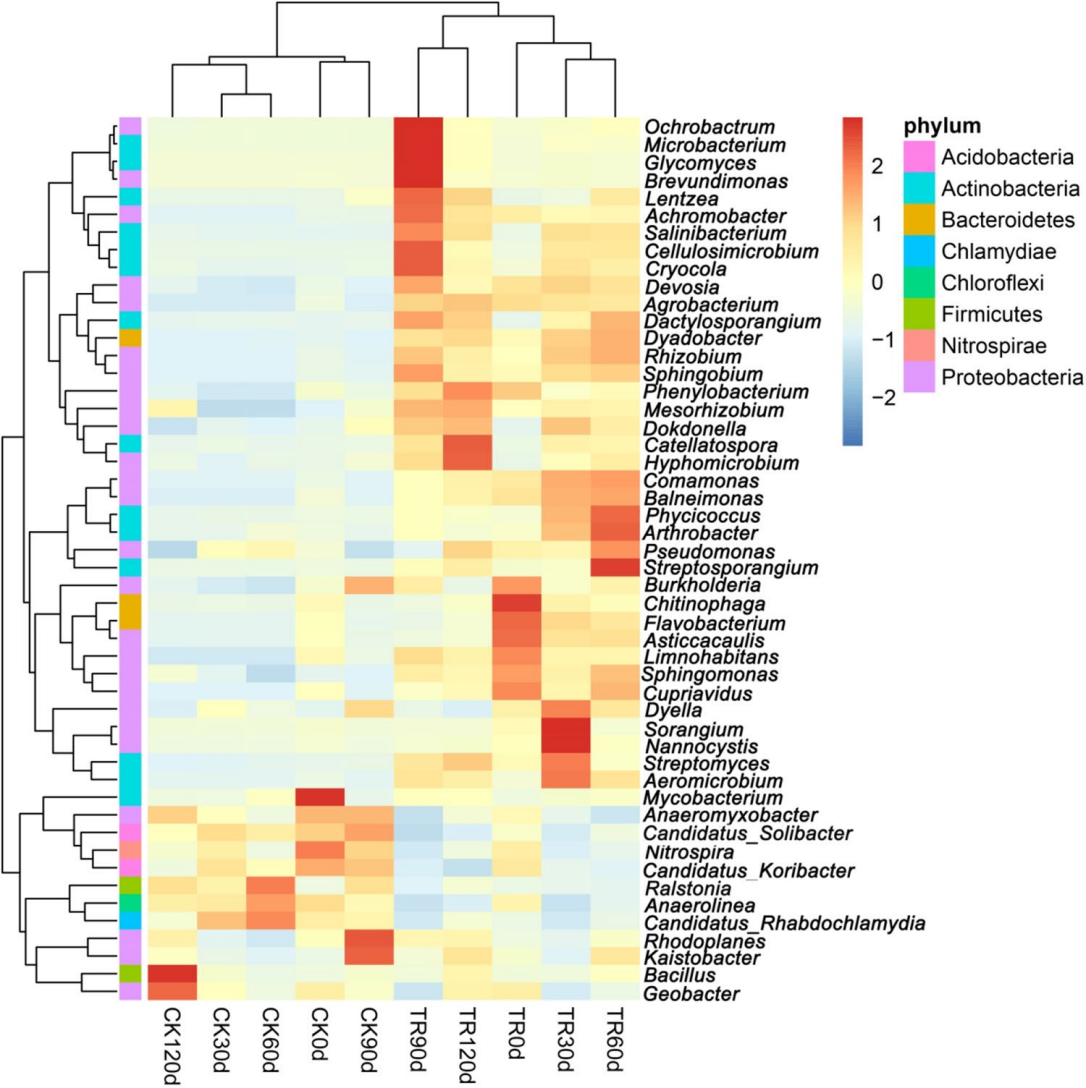
Dendrogram and heatmap of bacterial distributions of the top 50 abundant genera present in the bacterial community of the ten soil samples. The phylogenetic trees were calculated using neighbor-jointing method. The heatmap plot depicted the relative abundance of different soil samples within each genus (variables clustering on the vertical axis). The relative value of each genus was indicated by color intensity.

A hierarchically clustered heatmap was prepared based on the top 50 abundant bacterial communities at the genus level. As shown in Fig. 4, the TR and CK soil samples were separately classified into two groups. The heatmap plot also revealed the differences in genus abundance among the test soils. All the predominant genera in the TR samples were rare in the CK. Under the treatment of MRS, the relative abundances of *Ochrobactrum*, *microbacterium*, *Glycomyce*s, *Brevundimonas*, *Catellatospora*, *Hyphomicrobium*, *Phycicoccus*, *Arthrobacter*, *Streptosporangium*, *Chitinophaga*, *Sorangium*, *Nannocystis*, etc. were significantly increased. The relative abundance of *Ralstonia* (the pathogen genus of BW) was significantly decreased by MRS from 2.98% (CK) to 1.08% (TR) (*P* < 0.01). The relative abundances of soil bacteria were changed at different time periods both in TR and CK. For example, the relative abundance of *Ralstonia* in CK was increased first and then decreased, with the highest abundance in CK60d.

### Relationship among bacterial communities, soil properties and the severity of BW

**Figure 5:**
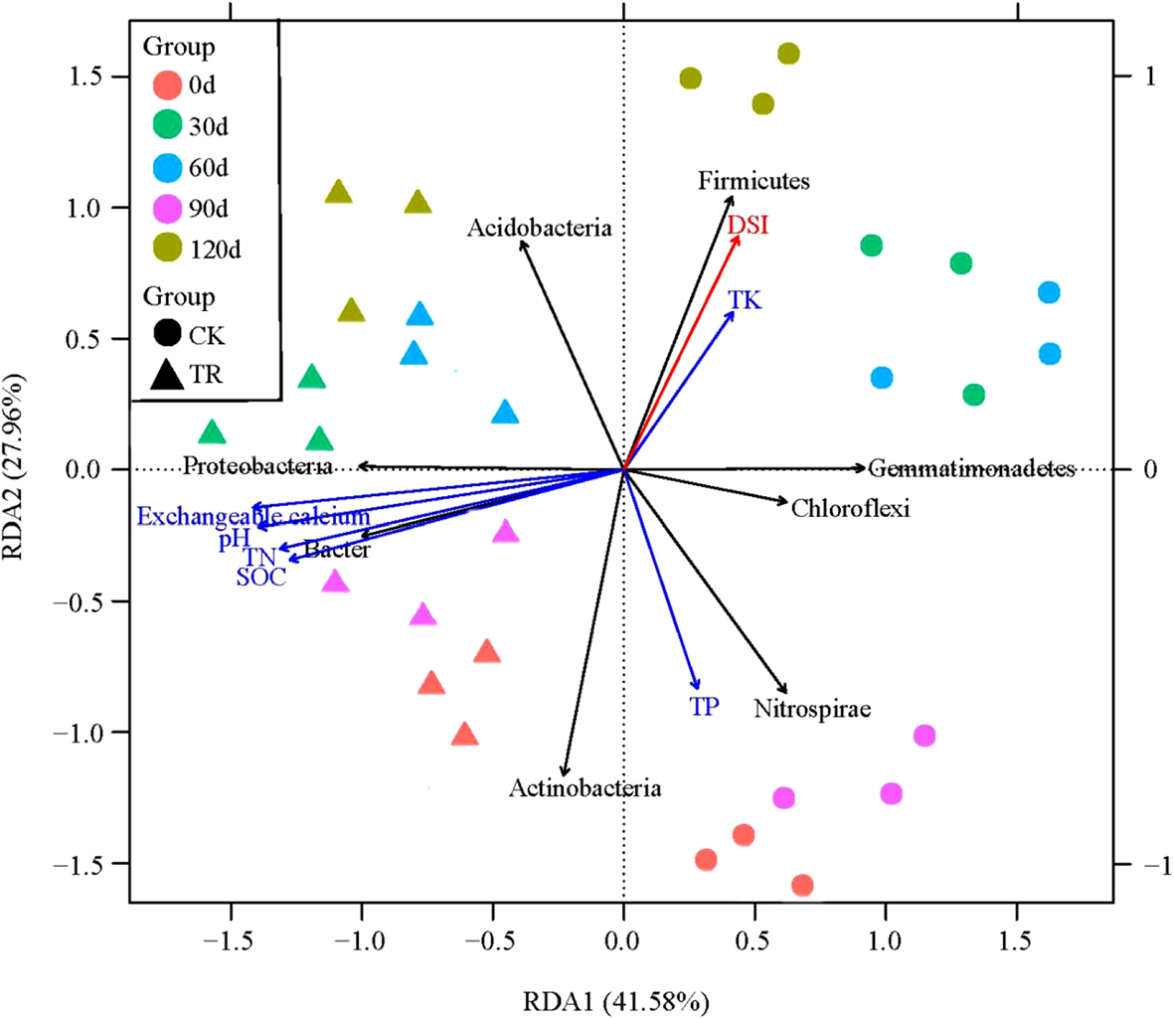
Redundancy Analysis (RDA) ordination plots show the relationships among bacterial phyla (black arrow), soil properties (blue arrow) and bacterial wilt disease severity (red arrow). Arrows indicated the direction and magnitude of variables.

A redundancy analysis (RDA) was performed to explore the relationship among the rhizobacterial communities, soil physicochemical properties and the severity of BW (Fig. 5). The first two axes of RDA explain 50.66% and 20.89% of the total variation in the data. The results of RDA demonstrated that the relative abundances of Bacteroidetes and Proteobacteria were positively correlated with pH, SOC, TN and exchangeable calcium concentrations and were negatively correlated with TK concentrations. In contrast, Firmicutes abundance was negatively correlated with pH, SOC, TN and exchangeable calcium and was positively correlated with TK concentrations. It was also indicated that the DSI of tomato BW was negatively correlated with the relative abundance of Actinobacteria (r^2^ = 0.8918), Bacteroidetes (r^2^ = 0.8350) and Proteobacteria (r^2^ = 0.8912) and was positively correlated with those of Gemmatimonadetes (r^2^ = 0.5758), Firmicutes (r^2^ = 0.6799) and Acidobacteria (r^2^ = 0.7485).

### Potential functional capacities of bacterial communities

**Figure 6:**
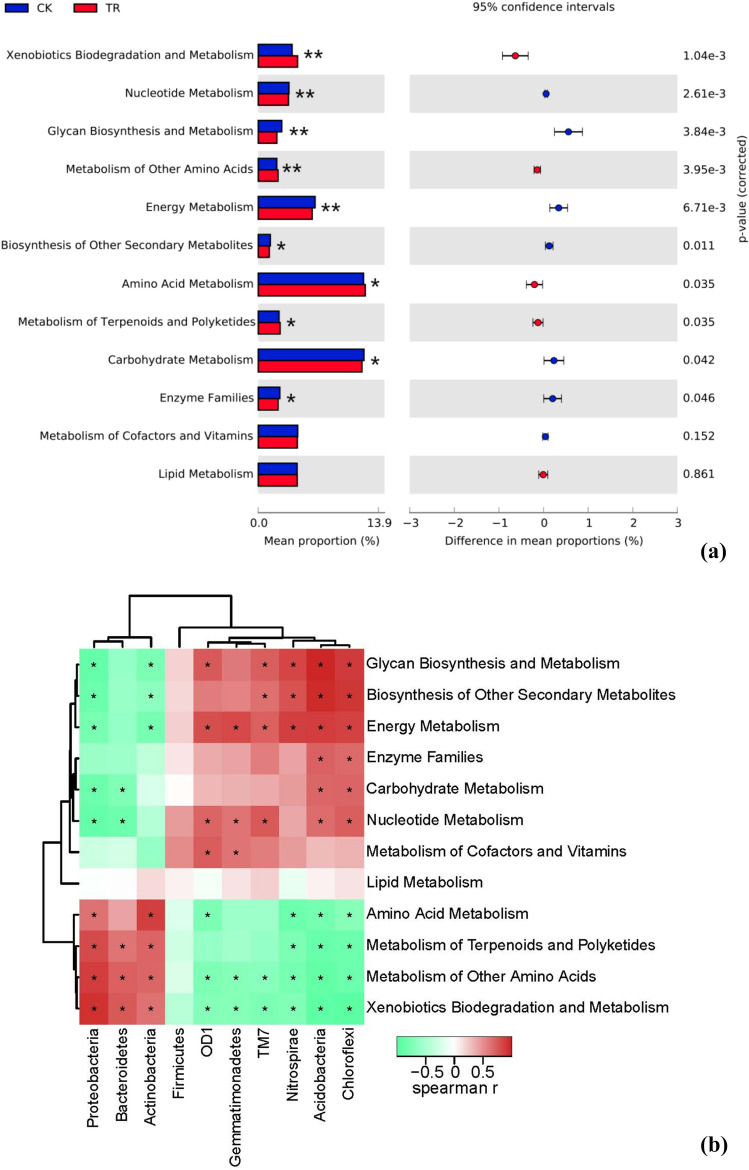
Potential metabolic functions of tested samples in KEGG pathways (levels 2) (**a**) and the correlation of metabolic functions and rhizobacteria (**b**). Heatmap depicts the correlation of rhizobacteria and metabolic functions at the phylum level. Red and green cells indicate positive and negative correlations, respectively. *P < 0.05, **P < 0.01.

As each bacterial species possesses specific KEGG metabolic pathways, the functional potentials of the bacterial community can be predicted^28^. Significant differences in the predicted KEGG functional pathways were found in the TR and CK treatments (Fig. 6a). Among 12 metabolic pathways, the highest relative abundance was carbohydrate metabolism (10.53% for TR, 10.76% for CK) and amino acid metabolism (10.87% for TR, 10.71% for CK), whereas the biosynthesis of other secondary metabolites had the least abundance (1.12% for TR, 1.24% for CK). Compared to the CK, the application of MRS increased the abundance of xenobiotic biodegradation (*p*-value 0.001) and amino acid metabolism (*p*-value 0.035); these soils also decreased the abundance of glycan biosynthesis and metabolism (*p*-value 0.004), enzyme families (*p*-value 0.046), energy metabolism (*p*-value 0.007), carbohydrate metabolism (*p*-value 0.042), and the biosynthesis of other secondary metabolites (*p*-value 0.011) (Fig. 6a).

The classical Spearman correlation analysis was carried out to estimate the correlation of bacteria and metabolic pathways at the phylum level (Fig. 6b). All bacterial phyla detected (except the phylum Firmicutes) were significantly correlated with the metabolism of other amino acids, and xenobiotic biodegradation and metabolism. Actinobacteria, Bacteroidetes and Proteobacteria had positive correlation with metabolism of other amino acids, and xenobiotic biodegradation and metabolism, but Acidobacteria, Chloroflexi, Gemmatimonadetes, Nitrospirae, OD1 and TM7 negative. Glycan biosynthesis and metabolism, biosynthesis of other secondary metabolites and energy metabolism were all significantly positive correlated with Acidobacteria, Chloroflexi, Nitrospirae and TM7, but significantly negative correlation with Actinobacteria and Proteobacteria (*P* < 0.05). Carbonhydrate metabolism was significantly positive correlation with Acidobacteria and Chloroflexi, but negative correlation with Bacteroidetes and Proteobacteria. Enzyme families were only significantly correlated with two out of ten bacterial phyla (Acidobacteria and Chloroflexi). Lipid metabolism had no significant correlation with any of the tested bacterial phyla.

## Discussion

This study confirmed that MRS possessed the potential to improve soil-related obstacles caused by the continuous cropping of tomatoes. MRS suppressed the disease severity index of tomato BW and had an average control efficiency of 83.52%. Moreover, large variance in physicochemical and microbial properties in the rhizosphere soil of tomato plants was observed following the application of MRS. This study provides a new practical method for soil restoration and provides a creative insight into the mechanisms involved in overcoming continuous cropping obstacles.

Knowing the characteristics of soil physicochemical properties with and without MRS could be helpful to understand the restorative mechanisms of MRS. Compared to the CK, the use of MRS significantly increased the value of soil pH, as well as SOC, TN, TP and exchangeable calcium contents. This result was consistent with that of previous studies in which soil pH, TN, TP and organic carbon content were sharply increased with biochar application^14,31^. However, the TK concentration had no significant influence by the application of MRS (except for 60 days samples), which was contrary to a previous report from Biederman and Harpole, who found that soil potassium concentration increased following biochar application^32^. Additionally, the contents of SOC, TN, TP and exchangeable calcium in TR decreased from seedling stage to havest stage. For example, SOC, TN, TP and exchangeable calcium contents were increased from TR 30 days to TR 120 days by 9.14%, 27.59%, 33.33% and 5.75%, respectively. This maybe because that (1) the plants in TR grew vigorously by significantly increasing root activity and plant height might contribute to nutrient uptake and transport; and (2) the fertilizer and MRS were applied only once throughout the tomato growth period, excepted for K fertilizer being added at fruit setting and ripening stage.

Soil pH is an important indicator of soil health. As reported, the low soil pH is positively correlated with the epidemic of BW^16^. The soil pH in healthy soils (6.20) was significantly higher than the BW infected soils (4.67)^33^. Li and Dong had reported that soil respiration with rock dust increased the original soil pH from acidic (4.81) to neutral^15^. In this study, the soil pH was increased by MRS more than 2 units, which indicated that this type of soil amendment could adjust soil pH and keep it in a nearly neutral environment. Evidence is mounting that pH, organic carbon, alkaline nitrogen, available potassium, and available phosphorus are negatively correlated with the severity of soil-born diseases^14,33–36^. Our data also showed that DSI was negatively correlated with soil pH, SOC, TN, TP and exchangeable calcium concentrations. High SOC, TN, TP and exchangeable calcium contents in the soil could increase the competitive capability of beneficial microorganisms against plant pathogen via nutrition competition^14^. The beneficial microorganisms could proliferate and attain cell densities above a specific threshold density at a time and place that is critical for pathogen infection^37^. Therefore, the results suggested that the application of MRS could create an environment favorable for tomato but unfavorable for *R. solanacearum*.

Soil microorganisms are very important for soil functions^38^. In this study, a more detailed focus on the bacterial communities revealed that MRS altered the microbial composition. The application of MRS increased the richness and diversity of bacteria in the tomato rhizosphere. A large body of literature illustrated that the richness and diversity of the soil microbial community is positively correlated with plant health^39,40^. Moreover, the addition of MRS increased the relative abundance of Proteobacteria, Actinobacteria and Bacteroidete*.* Proteobacteria is known to play important roles in carbon, nitrogen, and sulfur cycling^41^. Actinobacteria is a kind of heterotrophic bacteria, which plays a major role in the decomposition of soil organic matter^42,43^, and producing antibiotics to suppress various plant disease^44^. Bacteroidetes is very important as an indicator of soil health^2^ and was reported to possess the potential ability for biocontrol^45^. Yu et al. suggested that most microbes of the phylum Bacteroidetes could decompose cellulose and other hardly degradable aromatic compounds, which are very important in soil mineralisation^46^. However, the abundance of some phyla, such as Acidobacteria, were reduced. According to our results, this decrease of Acidobacteria was associated with the higher soil pH, since the bacteria in this phylum are usually acidophilic^47^.

Many studies have shown that soil physicochemical properties played very important roles in microbial community structure^48,49^. It had been reported that soil properties directly affected the microbial community and indirectly affected plant disease infection through microbial diversity, composition, and network interactions^50^. According to the RDA analysis, a range of correlations among rhizobacterial communities, soil physicochemical properties and DSI were observed. The relative abundances of Bacteroidetes and Proteobacteria were positively correlated with SOC, TN and exchangeable calcium concentrations, indicating that these two bacterial phyla prefer the enriched soil environments and belong to the eutrophic groups. Similar results had been reported previously^51–53^. Moreover, the relative abundances of Actinobacteria, Bacteroidetes and Proteobacteria were negatively correlated with the severity of tomato BW, suggesting that the bacteria of these phyla might be important in the suppression of bacteria wilt disease. For an example, several members in the phylum Actinobacteria are often considered as plant-beneficial microbes, which are important for maintaining ecosystem stability in fields with healthy plants^50^.

Previous studies indicated that nitrogen and carbon metabolism can influence or be influenced by the structure of microbial communities^54,55^. In this study, the functional capabilities of bacterial communities in tomato rhizosphere fractions were compared with and without MRS treatments. According to the KEGG pathway, the application of a MRS increased the abundance of some nitrogen metabolism (e.g. amino acid metabolism) and reduced the abundance of carbon metabolism (e.g. glycan biosynthesis and metabolism). The amino acid metabolism have been reported to enhanced microbial growth and activity by suppling more carbon, nitrogen, and energy sources for microbes^56^. In this study, the amino acid metabolism was significantly positive with the relative abundance of Proteobacteria and Actinobacteria.

## Conclusions

The application of MRS could reduce the severity of tomato BW, increase root activity and plant yield. This capacity was associated with changes in soil physicochemical properties as well as the rhizobacterial community. The relative abundances of some phyla (e.g., Actinobacteria) were increased by MRS, whereas those of other phyla (e.g., Acidobacteria) were decreased. In conclusion, the application of MRS can re-establish the soil bacterial community and is beneficial to plant health.

## Supplementary information

Supplementary information
