# Supplementary material for: Effects of a microbial restoration substrate on plant growth and rhizosphere bacterial community in a continuous tomato cropping greenhouse
Source: Sci Rep. 2020 Aug 13;10:13729. doi: 10.1038/s41598-020-70737-0 (PMC7426824; doi:10.1038/s41598-020-70737-0)
Supplement: Supplementary file 1 — Supplementary information [file 41598_2020_70737_MOESM1_ESM.docx]

**Scientific Reports**

**Title: Effects of a microbial restoration substrate on plant growth and rhizosphere bacterial community in a continuous tomato cropping greenhouse**

Authors: Xuefang Zheng^1^, Ziran Wang^2^, Zhu Yujing^1^, Jieping Wang^1^, Bo Liu^1*^

(^1^Agrobiological Resource Research Institute, Fujian Academy of Agriculture Sciences, Fuzhou 350003, China; ^2^Dean of Department of Biochemistry and Biotechnology, School of Life Sciences, Xiamen University, Xiamen, 361102, China)

*Corresponding author

Bo Liu*, fzliubo@163.com; Tel.:+86-0591-87863227

**Table S1.** Correlation coefficients between soil properties and plant biological characteristic

| Items | pH | SOC | TN | TP | TK | Exchangeable calcium | Plant height | Root activity | Yield |
| --- | --- | --- | --- | --- | --- | --- | --- | --- | --- |
| pH | 1 |  |  |  |  |  |  |  |  |
| SOC | 0.998** | 1 |  |  |  |  |  |  |  |
| TN | 0.965* | 0.978** | 1 |  |  |  |  |  |  |
| TP | 0.576 | 0.606 | 0.727 | 1 |  |  |  |  |  |
| TK | -0.998** | -0.994** | -0.949* | -0.521 | 1 |  |  |  |  |
| Exchangeable calcium | 0.999** | 0.999** | 0.970* | 0.592 | -0.996** | 1 |  |  |  |
| Plant height | 0.981** | 0.989** | 0.997** | 0.707 | -0.967* | 0.985** | 1 |  |  |
| Root activity | 0.988** | 0.993** | 0.985** | 0.693 | -0.976* | 0.991** | 0.995** | 1 |  |
| Yield | 0.997** | 0.997** | 0.971* | 0.630 | -0.991** | 0.998** | 0.987** | 0.996** | 1 |

* correlation is significant at the 0.05 level and ** correlation is significant at the 0.01 level.

| 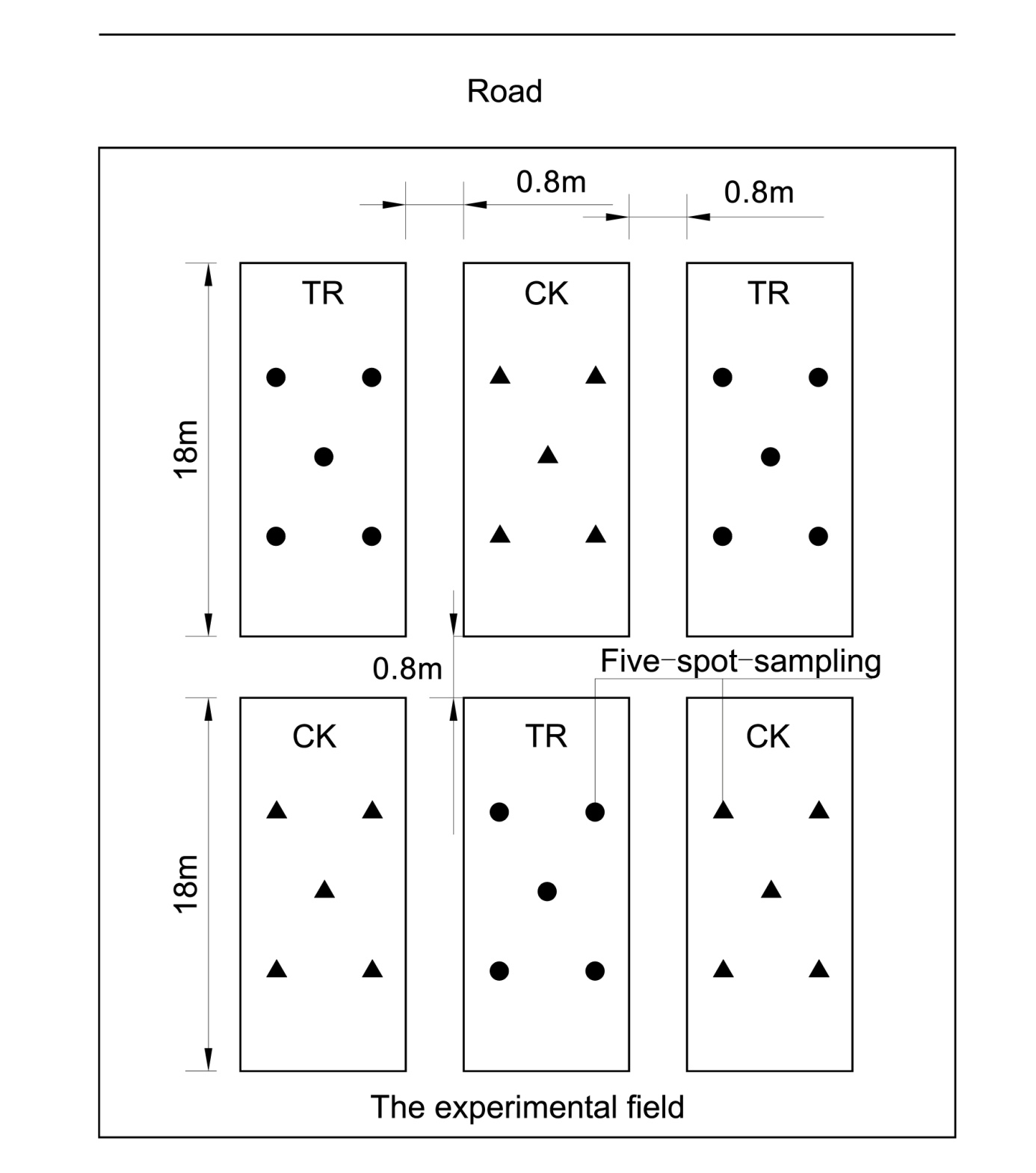 |
| --- |
| **SuSupplementary Fig.S1.** Diagram of different treatment plots in the field. “TR” represents microbial restoration substrate treatment; “CK” represents non- microbial restoration substrate treatment. Five solid circles in each plot of TR and five solid triangles in each plot of CK represent of five-spot-sampling of rhizosphere soils for TR and CK, respectively. |
